# Report of an Italian family carrying a typical Indian variant of the Nilgiris tribal groups resulting from a *de novo* occurrence

**DOI:** 10.1038/hgv.2017.57

**Published:** 2018-01-04

**Authors:** Giulia Canu, Giorgia Mazzuccato, Andrea Urbani, Angelo Minucci

**Affiliations:** 1 Department of Diagnostic and Laboratory Medicine, Institute of Biochemistry and Clinical Biochemistry, ‘Agostino Gemelli’ Foundation, Rome, Italy; 2 Proteomics and Metabolomics Unit, IRCCS- ‘Santa Lucia’ Foundation, Rome, Italy

## Abstract

G6PD deficiency is quite common in Italy where it is characterized by extreme molecular and biochemical heterogeneity. We report a 15-year-old Italian boy with *G6PD Nilgiri (c.593G>A,* p.Arg198His), a typical Indian variant of the Nilgiris tribal groups. Further, this variant was biochemically characterized, and the molecular screening of the family highlighted a *de novo* mutational event. To date, this family is the first Caucasian family carrying the *G6PD Nilgiri* variant.

Although it is difficult to detect G6PD-deficient patients given that affected people are asymptomatic until they are exposed to triggers, >400 million individuals are thought to be G6PD-deficient, exhibiting high genetic heterogeneity and making this enzymopathy the most common clinically significant enzyme defect.^1^

G6PD deficiency occurs most frequently in Africa, Asia, the Mediterranean and the Middle East, synchronizing with endemic malaria.^2^ In fact, the prevalence of G6PD deficiency correlates with the geographical distribution of malaria, leading to the postulate that G6PD deficiency provides partial protection against this infection.^3^

To date, >200 different *G6PD* pathogenic variants have been identified worldwide, and each ethnic population exhibits a peculiar mutation profile. All *G6PD* variants are classified from Class I to V according to the residual enzyme activity and type of clinical manifestations.^4,5^

In 2008, Chalvam *et al.*^6^ reported the screening results for G6PD deficiency among 1,125 male individuals from different tribal groups of the Nilgiri district in Southern India. A *hitherto* unreported *G6PD* variant was identified in four individuals. This new variant (*c.593G>A,* based on NM_001042351.2, rs137852332) causes a predicted amino acid change of arginine (Arg) to histidine (His) at codon 198 in exon 6. This variant was confirmed by a family study and was designated *G6PD Nilgiri.*

Chalvam indicates that further studies must be performed to determine the prevalence and distribution of this variant in different population groups. Moreover, Chalvam *et al.* was unable to classify this variant given that a sufficient blood sample was not available for biochemical characterization of the residual enzyme. To date, *G6PD Nilgiri* has never been reported in the literature again.

In Italy, G6PD deficiency is characterized by extreme molecular and biochemical heterogeneity; in addition to Sardinia and Sicily, where higher disease prevalence is present (from 2 to 15%), G6PD-deficient subjects are also found in other Italian regions, such as Campania, Basilicata, Puglia and Lazio.^7^ All these regions presented endemic malaria in the past.

At least 10 distinct *G6PD* point variants have been reported in Italy,^7^ and >94% of those (based on NM_001042351.2 reference) include *c.563C>T* (*G6PD Mediterranean*), *c.844G>C* (*G6PD Seattle*) *c.202G>A/c.376A>G* (*G6PD A*^*−*^*), c.1003G>A* (*G6PD Chatam*) and *c.1347G>C* (*G6PD Cassano*). Thus, we are only able to perform preliminary mutational scanning of these variants.^8^ Moreover, novel and rare *G6PD* variants are also present.^9^

We report a case of an asymptomatic 15-year-old male born to parents of Italian descent (Campania region) with severe G6PD deficiency discovered during military recruiting (Table 1). He had no relevant family history. After written informed consent was provided, direct sequencing of the entire *G6PD* gene was performed given that the patient was negative for the most common Italian variants. The patient was hemizygous for the *G6PD Nilgiri* variant.

The family study confirmed the maternal inheritance of the variant (Figure 1a). Conversely, the p.Arg198His variant was not identified in the patient's grandparents, from whom DNA from buccal cell samples was analyzed (Figure 1b). The *G6PD* intragenic markers *c.1365-13T>C* (rs2071429) and *c.1311C>T* (rs2230037) were informative of the mother-grandparents relationships. These findings suggested that the *G6PD Nilgiri* variant in this family was a result of a *de novo* mutational event.

*G6PD Nilgiri* involves the same codon 198 that is mutated in *G6PD Coimbra* (*c.592C>T*, p.Arg198Cys). However, in *G6PD Coimbra,* the mutation is at nt 592, which is the first base of the codon, whereas the *G6PD Nilgiri* variant is located at the second base of the same codon. *G6PD Coimbra* is very close to the *G6PD Mediterranean* variant within exon 6 and has similar kinetic properties, namely high affinity for G6P and NADP^+^ and a high rate of deamino-nicotinamide adenine dinucleotide phosphate (dNADP) and 2-deoxy glucose-6-phosphate utilization compared with the G6PD-B enzyme (considered the normal phenotype). It has been suggested that the region encompassing the *Coimbra* and *Mediterranean* variants is spatially close and involved in the enzyme’s interactions with its substrate. Both variants are classified as *Class II*.^4^

This study reports for the first time the *G6PD Nilgiri* in Italy, and we were able to definitively classify this variant as *Class II* based on both on the residual enzyme activity and the clinical manifestations of the patient and his family members. In addition, this case provides further evidence on the prevalence and distribution of the *G6PD Nilgiri* in different population groups, confirming the high genetic heterogeneity of the G6PD deficiency in Italy,^7^ although owing to a *de novo* occurrence. In this context, we underscore c*.592C>A* (p.Arg198Ser)^10^ as an additional variant, indicating that the mutation at the same nucleotide site results from a *de novo* event; this consideration may suggest that this region is prone to such molecular events.

In conclusion, we highlight four main points: (a) a clinical picture of the patient and residual enzymatic activity are of primary importance for the definitive classification of each *G6PD* variant; (b) sequencing of the entire *G6PD* gene is mandatory especially in those patients from peculiar Italian regions; (c) the use of next-generation sequencing in G6PD-deficient subjects to diagnose common, rare and novel *G6PD* variants is now suggested,^11^ and finally, (d) *G6PD* is confirmed to be a gene prone to *de novo* events, as reported.^12–17^

## Additional information

**Publisher’s note:** Springer Nature remains neutral with regard to jurisdictional claims in published maps and institutional affiliations.

## Figures and Tables

**Figure 1:**
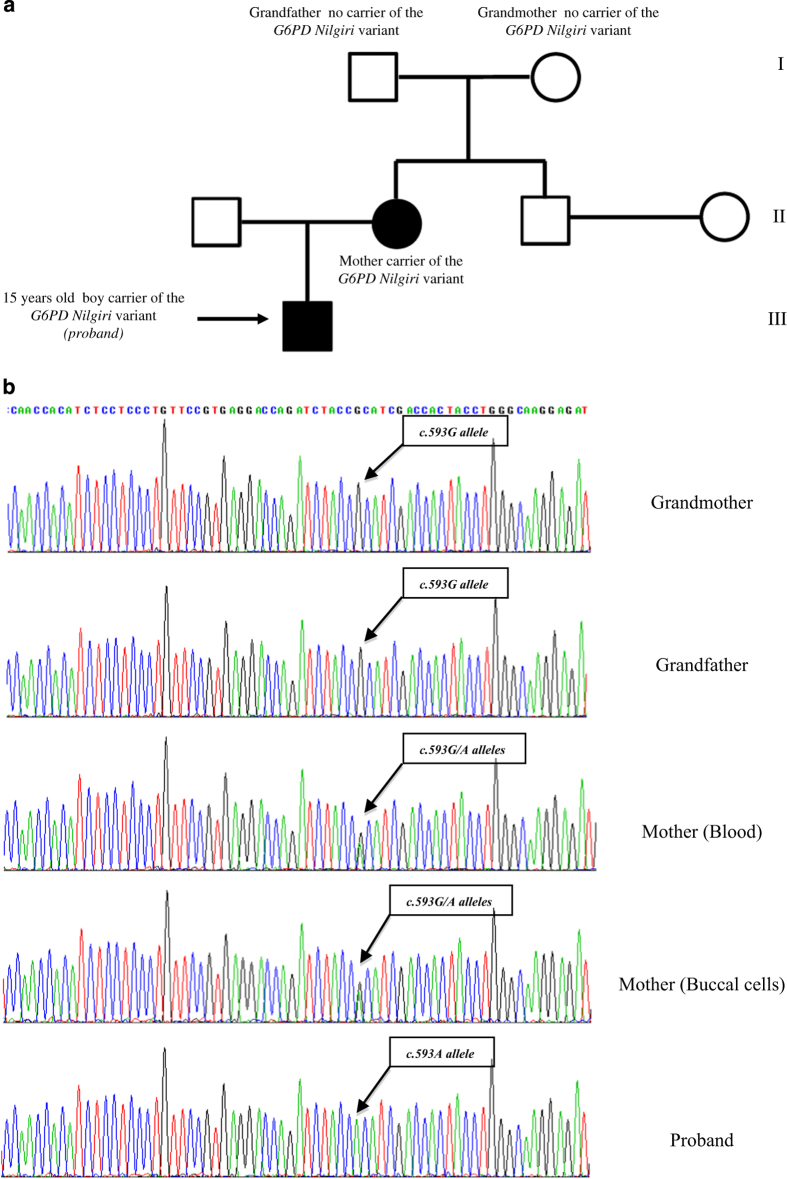
(**a**) The pedigree of the patient’s family is showed; (**b**) Sequences for the *G6PD Nilgiri* of proband and his relatives are showed. For the proband’s mother mutant and normal alleles were found in both hematopoietic and buccal cells. The absence of the *c.593A* allele in the grandparents of the proband shows a *de novo* occurrence of the *G6PD Nilgiri* in this family.

**Table 1:** G6PD activity, some hematological values and molecular results of proband and his relatives

	*Proband*	*Mother*	*Grandmother*	*Grandfather*	*Reference values*
G6PD activity^a^	0.1	—	—	—	9.2–13.8 U/gHb
G6PD/6PGD^b^	—	0.4	—	—	>0.85
Hemoglobin	14.5	12.8	—	—	12.2–16.6 g/dl
RBCs^c^	4.25	4.46	—	—	4.20–5.60×10^12^/l
*G6PD Nilgiri*	Hemizygote^d^	Heterozygote^d^	WT^e^	WT^e^	

a G6PD biochemical activity was performed using a commercial Kit (Sentinel diagnostics, Milano, Italia).

b G6PD/6PGD was evaluated using a commercial kit (NUREX diagnostics, Sassari, Italy) able to detect females *G6PD* variants carriers.

c RBCs: red blood cells.

d Molecular testing performed on DNA from peripheral blood.

e Molecular testing performed on DNA from buccal swab.
